# Management of Hypertensive Crises in Children: A Review of the Recent Literature

**DOI:** 10.3389/fped.2022.880678

**Published:** 2022-04-15

**Authors:** Nicola Bertazza Partigiani, Rachele Spagnol, Laura Di Michele, Micaela Santini, Benedetta Grotto, Alex Sartori, Elita Zamperetti, Margherita Nosadini, Davide Meneghesso

**Affiliations:** ^1^Paediatric Nephrology Unit, Department of Womens's and Children's Health, University Hospital of Padua, Padua, Italy; ^2^Paediatric Neurology and Neurophysiology Unit, Department of Womens's and Children's Health, University Hospital of Padua, Padua, Italy

**Keywords:** hypertension, hypertensive crisis, hypertensive emergency, beta-blockers, calcium-channel blockers, children, pediatric

## Abstract

Hypertensive emergency is a life-threatening condition associated with severe hypertension and organ damage, such as neurological, renal or cardiac dysfunction. The most recent guidelines on pediatric hypertension, the 2016 European guidelines and the 2017 American guidelines, provide recommendations on the management of hypertensive emergencies, however in pediatric age robust literature is lacking and the available evidence often derives from studies conducted in adults. We reviewed PubMed and Cochrane Library from January 2017 to July 2021, using the following search terms: “hypertension” AND “treatment” AND (“emergency” OR “urgency”) to identify the studies. Five studies were analyzed, according to our including criteria. According to the articles reviewed in this work, beta-blockers seem to be safe and effective in hypertensive crises, more than sodium nitroprusside, although limited data are available. Indeed, calcium-channel blockers seem to be effective and safe, in particular the use of clevidipine during the neonatal age, although limited studies are available. However, further studies should be warranted to define a univocal approach to pediatric hypertensive emergencies.

## Background

A hypertensive crisis is defined as an acute severe elevation in blood pressure (BP). There is not an absolute threshold to define severe hypertension. The European Society of Hypertension (ESH) guidelines suggest that a value of 20% above the stage 2 hypertension limit may indicate a critical point for severe hypertensive crisis in children (1). According to the guidelines published by the American Academy of Paediatrics (AAP), clinicians should be concerned about the development of target organ damage when a child's BP increases 30 mmHg or more above the 95th percentile for age and height (2, 3).

Depending on the degree of BP increase and the presence of acute end-organ damage, a hypertensive crisis can be further classified as a hypertensive emergency or urgency (4). A hypertensive emergency is a life-threatening condition associated with severe BP elevation and organ damage, such as neurological, renal or cardiac dysfunction (5–7). Children can manifest headache, confusions, seizures, nausea or vomiting, visual symptoms and facial palsy (8–10). On the other hand, a hypertensive urgency is defined as a sudden severe hypertension without organ damage and it does not represent a life-threatening condition (5, 11–13).

Most children who experience hypertensive crises have secondary hypertension, most commonly due to kidney disease (14–18). In a study conducted at Great Ormond Street Hospital, the most common cause of severe hypertension was chronic kidney disease including reflux nephropathy, glomerular disease, renovascular disease, obstructive uropathy and haemolytic-uremic syndrome, which together accounted for 76% of the cases (8). In a more recent study on children with severe hypertension, etiologies included kidney transplantation complications, multiorgan failure, renovascular disease and acute renal failure (19–21). Thus, patients who present with acute severe hypertension should be expediently evaluated for secondary causes (2, 22).

### Treatment

Hypertensive crises are not common in children, however they represent an emergency that must be promptly treated to prevent irreversible damage of vital organs, including possible neurological and visual sequelae (3, 23, 24).

The ESH guidelines specify that patients presenting a hypertensive emergency should always be treated with intravenous drugs, preferentially administered in continuous infusion and not by bolus, to reduce the risk of hypoperfusion (1). According to the AAP guidelines, however, treatment should be started with enteral drugs if the patient is able to tolerate oral therapy and if life-threatening complications have not yet developed. In fact, intravenous agents should only be used when oral administration is not possible due to the patient's clinical status or when a serious complication has already developed (2).

Both AAP and ESH groups agree that BP should be reduced by no more than 25% of the desired target reduction in the first 8 h; complete normalization of blood pressure should be completed in the subsequent 12–24 h (1, 2).

Sodium nitroprusside appears to be the most widely used first-line agent for hypertensive crises. However, its relative contraindication in children with renal or hepatic disease and its risk to cause cyanide toxicity with prolonged use are a cause for concern (25–27).

There is a lack of robust evidence to guide the evaluation and management of children with acute presentations of severe hypertension. Most of the available data derives from studies conducted in adults; acute severe hypertension infrequently affects pediatric patients and, consequently, data on the efficacy and safety of the majority of antihypertensive agents, as well as the associated adverse events, are very limited in this population.

This review aims to analyse the recent evidence on the pharmacological treatment of pediatric hypertensive crisis; in particular the goal is to identify the drug with the best efficacy and safety profile for the first-line treatment of hypertensive emergencies and urgencies.

## Methods

We conducted a review on Medline (https://pubmed.ncbi.nlm.nih.gov) and Cochrane Library selecting studies about the efficacy and safety profile of pharmacological treatments of hypertensive urgencies and emergencies from January 2017 to September 2021.

We used the keywords “hypertension” AND “treatment” AND (“emergency” OR “urgency”) to identify the studies. All potentially relevant titles and abstracts were retrieved and assessed for eligibility by seven investigators independently. We excluded studies based on design (case report, review and systematic review, meta-analysis, animal models or editorials), or patient populations (patient > 18 years). We included randomized controlled trials or observational studies, both prospective and retrospective, with more than ten eligible patients.

Our study conforms to the Preferred Reporting Items for Systematic Reviews and Meta-analysis guidelines (28).

The full text of each of the selected articles was manually reviewed by three investigators (N.B.P, L.D.M., and R.S.) who were not blinded to the journal name, institution or study authors.

## Results

**Figure 1:**
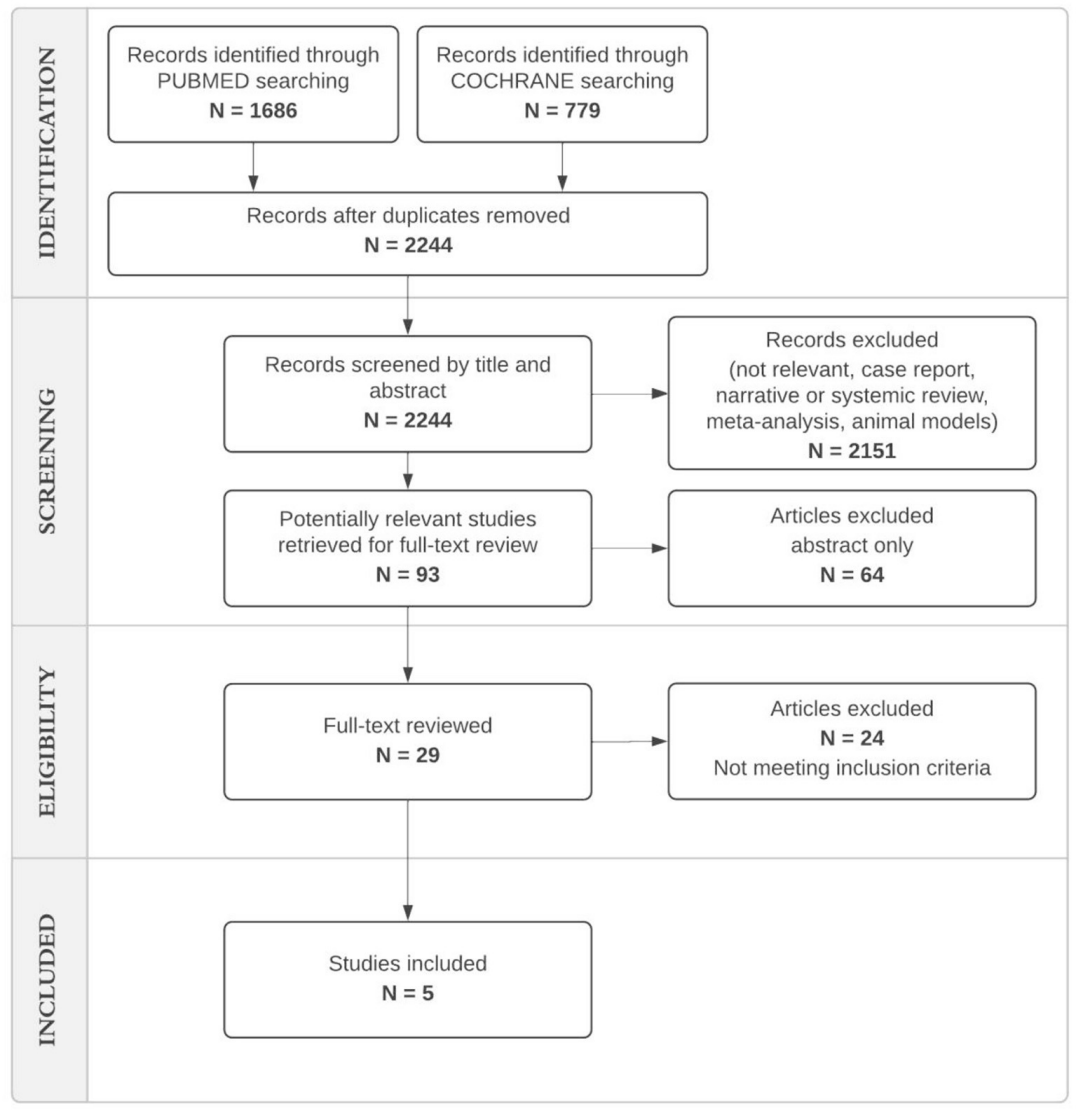
Flow chart: identification of studies via Pubmed and Cochrane Library.

The search retrieved a total of 1,686 references from Medline and 779 from Cochrane Library. After screening the titles and abstracts, we identified 29 studies for full-text review. Following a full-text review we identified a total of five eligible studies meeting our inclusion criteria (5, 25, 29–31) (Figure 1). The selected studies are reported in Table 1. All studies had a retrospective design and were conducted between 2008 and 2019. The study setting was the pediatric intensive care unit (PICU) in four of the five studies (Saqua, Lad, Wu, Stone).

**Table 1:** Studies meeting inclusion criteria and their characteristics.

**References**	**Journal**	**Country**	**Study design**	**Setting**	**Time period**	**No. of children**	**Age range (mean age)**	**Type of hypertension**	**Treatment**	**Drug**	**Objective**	**Efficacy**	**Safety**
Saqan and Thiabat (5)	Pediatr Nephrol	Jordan	Retrospective	Single-center PICU	2008–2015	13	2 month−16 y (8.25)	Hypertensive emergency	Pharmacological	b-blocker Metoprolol	Efficacy of iv metoprolol in hypertensive emergency	BP <90th for their age and height in 100% patients	Not reported
Lim et al. (29)	J Pediatr Intensive Care	Singapore	Retrospective	Single-center Tertiary Pediatric Hospital	2009–2015	37	1 month−21 y (12.4)	Hypertension emergency and urgency	Pharmacological	CCB, b-blocker Nifedipine, Labetalol	(1) First treatment in hypertensive crisis (2) Outcomes	(1) Nifedipine oral 62.1%; Labetalol iv 21.6% (2) Both effective treatment	Not reported
Lad et al. (25)	Indian J Pediatr	India	Retrospective	Single-center PICU	2009–2019	56	1 month−12 y (6.9–8)	Hypertension crisis	Pharmacological	b-blocker Labetalol vs. Nitroprusside/Nitroglycerin	Efficacy and safety of iv labetalol in hypertensive crisis	- BP control (<95th pct 12–48 h) in group with labetalol 62% vs. non-labetalol group 30.3% - Higher neurological recovery in labetalol group	Labetalol vs. non-labetalol groups hypotension 13 vs. 15% hyperkaliemia 0 vs. 0.03%
Wu et al. (30)	Pediatr Crit Care Med	USA	Retrospective	Single-center PICU	2016–2019	38	0.5–12 y (2.7)	Hypertension crisis	Pharmacological	CCB Clevidipine	Clevidipine iv for BP control in pediatric patients on mechanical circulatory support	- Efficacy in hypertension management - Cost - effective alternative compared to traditional short-acting agents	Hypotension 0% hypertriglyceridemia 9%
Stone et al. (31)	Ann Thorac Surg	USA	Retrospective	Single-center PICU	2010–2015	68	0–18 y (0.7)	Postoperative hypertension	Pharmacological	CCB Nicardipine	Safety of nicardipine as a first-line agent for BP control after cardiac operation	Anyone patients receiving nicardipine required cessation therapy, an additional drug, or transition to an alternative antihypertensive agent to achieve the target PO BP	Hypotension 13% NO major adverse events

*BP, blood pressure; CCB, calcium-channel blockers; IV, intravenous; NICU, neonatal intensive care unit; PICU, pediatric intensive care unit; SE, side effects*.

Overall, the five eligible studies included a total of 212 children (median 38 patients per study, range 13–68).

All the studies were monocentric and included patients with age range 0–18 years old.

The patients included in the studies suffered from hypertensive emergency and urgency (Saquan, Lim), hypertension crisis (Lad, Wu) and postoperative hypertension (Stone).

Primary outcome of all studies was to evaluate the efficacy of the antihypertensive therapy.

The cause of hypertensive crisis was identified in 212 patients. Main causes were cardiac surgery (32%), chronic kidney disease (28.7%) and patients undergoing mechanical circulatory support (17.9%). Less common causes were tumors (2.8%), pheochromocytoma (2.3%), aortic coarctation (0.9%) and iatrogenic hypertension (0.9% cases). Only four cases presented a hypertensive crisis in the course of essential hypertension (1.8%). In 18 patients (8.4%), the cause was not identified (Saquan) (Liam) (Lad).

Investigated antihypertensive drugs included beta blockers (labetalol, metoprolol, propranolol), calcium channel blockers (nifedipine), ACE-inhibitors (enalapril), diuretics (furosemide) and nitroglycerin (Saquan) (Liam) (Lad). A comparison between two classes of medications (labetalol and nitroglycerin) was performed only in Lad's study.

Both oral and intravenous antihypertensive drugs were analyzed in Lim et al. (29); other studies considered only intravenous administration (5, 25, 29–31).

In Lad et al. work, 56 pediatric intensive care unit (PICU) patients were divided into labetalol and not-labetalol treatment groups. The first included 23 children receiving labetalol infusion as first-line or add-on treatment or transition from other IV antihypertensive infusions (sodium nitroprusside, nitroglycerine) in the first 24 h of management. Non-labetalol group included 33 children who received sodium nitroprusside and/or nitroglycerine infusion for BP control. BP reduction was significantly higher in the labetalol group (62 vs. 30.3% of non-labetalol group, *p* = 0.03). In addition, the rate of neurological recovery at discharge was significantly higher in the labetalol group (56.6 vs. 18.7% of non-labetalol group, *p* = 0.02) (25).

Saqan and Thiabat considered 13 patients who underwent metoprolol infusion in PICU. The starting dose was 0.5 mcg/kg/min and was increased according to each patient's BP to a maximum dose of 5 mcg/kg/min. The percentage of systolic BP reduction after 24 h ranged between 15 and 44% of the initial value. All patients were discharged with a BP within the 90th percentile for their age and height. Twelve patients had no neurological sequelae. In this study metoprolol was effective in lowering BP safely, regardless of the etiology of hypertension (5).

Lim et al. analyzed 37 patients who were treated for hypertensive crises in a tertiary pediatric hospital from 2009 to 2015. The choice for initial therapy was between oral nifedipine, oral propranolol, oral enalapril, intravenous labetalol and intravenous furosemide. Oral nifedipine was the most commonly used antihypertensive medication for the initial management of our patients with hypertensive crisis (59.5%). The most frequently administered intravenous drug was labetalol (21.6%). Intravenous furosemide was the first choice for all patients with hypertensive crisis secondary to post-streptococcal glomerulonephritis. No significant statistical differences were found between hypertensive urgency and emergency treatment groups in terms of efficacy. The study concludes that oral nifedipine and intravenous labetalol are both effective treatments (29).

Stone et al. enrolled 68 children undergoing cardiac surgery at the University of Virginia from 2010 to 2015, who received nicardipine for the management of postoperative hypertension. None of the patients discontinued treatment because of adverse effects, required additional antihypertensive agents or transitioned to an alternative antihypertensive drug to achieve the target postoperative blood pressure (31).

Wu et al. reviewed a cohort of 38 patients in mechanical circulatory support (MCS) who received a total of 45 clevidipine infusions. The median age of the patients was 2.7 years and included neonates. Clevedipine was administered as first choice in 57.8% of cases. Twenty-six clevidipine infusions were administered as a single agent without sodium nitroprusside. Seven patients were switched from sodium nitroprusside to clevidipine to avoid cyanide toxicity and a majority of them had elevated serum creatinine. Eleven patients transitioned from clevidipine to enteral antihypertensive agents at PICU discharge (30).

Regarding the safety of the analyzed drugs, the most frequently described adverse effects overall were hypotension and hyperkalemia (25, 31).

In particular, Lad et al. observed hypotension in 13% of patients treated with labetalol and in 15% of patients treated with con nitroglycerin or nitroprusside. Nitroprusside was also associated to hyperkaliemia in 0.03% of patients (25). Patients treated with nicardipine infusion presented hypotension in 13% of cases (31). Clevidipine infusion brought an increase of triglycerides in 9% of patient (30). No side effects were described in Sequan's and Lim's studies.

## Discussion

Our review provides updated results on the pharmacological standard of care for pediatric emergency arterial hypertension.

Early treatment of hypertension crises prevents the development of complications in adulthood, such as cardiovascular diseases and organ damage.

Currently, there is no agreement on which drug is the most effective and safe to use as a first line in a hypertensive crisis. According to the European ESH guidelines, sodium nitroprusside and labetalol are the most commonly used drugs for hypertensive emergencies in children (1, 32). The AAP guidelines indicate as possible pharmacological agents esmolol, hydralazine, labetalol, nicardipine and nitroprusside for patients with life-threatening symptoms; while they point to clonidine, fenoldopam, hydralazine, isradipine and minoxidil as pharmacological agents for patients with less severe symptoms (2, 33).

Although not listed among the drugs recommended by the most recent guidelines, oral nifedipine was routinely used for the treatment of hypertensive emergencies, and administered in 50% of patients, in the retrospective study by Lim et al. (29). Oral nifedipine has been associated with an increased risk of renal, cerebral or coronary ischaemia in the adult population and is no longer considered acceptable for the initial treatment of hypertensive crisis (34). However, it has been and is still widely prescribed by pediatricians for the treatment of acute hypertension in children due to the efficacy in blood pressure control and its safety profile (34–36). The pediatric population may be better able to tolerate nifedipine as children typically do not have significant vascular and cardiovascular risk factors, unlike the adult population, which result in significant mortality and morbidity from large changes in end organ perfusion (35, 37).

Beta blockers appear effective and safe according to the recent studies reviewed. Lad et al. (25) showed that labetalol was more effective in BP reduction compared to nitroprusside, chosen as standard of treatment, with similar incidence of side effects. This is the first study comparing the efficacy of the two drugs and it demonstrates a superiority of labetalol over nitroprusside, despite the strong limitations due to the small cohort size and the retrospective nature.

Accordingly, labetalol was the most utilized intravenous drug for the management of hypertensive crises among the analyzed group in the study by Lim et al. (29) and was deemed effective.

Metoprolol appeared safe and effective in the small pediatric cohort admitted to PICU for hypertensive crises reported by Saqan and Thiabat (5).

Interestingly, labetalol treatment was associated with a better neurological outcome than nitroprusside in the study by Lad et al. (25) and Saqan and Thiabat (5) reported that 92% of patients treated with metoprolol had no neurological sequelae.

A good neurological outcome can be the determining factor in the choice of antihypertensive therapy in terms of efficacy and patient management.

In this context, a main role is given by selective calcium-channel blockers. Among these drugs, the use of clevidipine is particularly interesting (30, 38).

The study by Wu et al. on patients admitted to the PICU in mechanical circulatory support, showed how the use of a clevidipine infusion was effective in the management of hypertension and not associated with hypotensive events even in neonatal populations. The use of clevidipine may be reasonable for BP management in pediatric patients on MCS, particularly those with kidney injury at risk of cyanide toxicity with SNP. Moreover, clevidipine is emulsified in fat and provides additional calories without a clear effect on serum triglyceride. Clevidipine is thought to be safe in this population because its effect is limited to peripheral calcium channels without involving central calcium channels and so preserving the myocardial function (30).

Stone et al. analyzed the efficacy of nicardipine, a selective calcium channel blocker, in the PICU management of hypertension after surgery, in particular in patients undergoing cardiac surgery, showing its safety and efficacy (31).

Therefore, we suggest beta blockers or calcium-channel blockers to be used as the first pharmacological line in case of hypertensive crisis, given the demonstrated efficacy and safety. In our review, no main side effects have been reported in the included studies. These drugs should in fact be preferred to the more commonly used sodium nitroprusside (5, 29).

Indeed, there are no recent studies regarding the safety of sodium nitroprusside in the pediatric hypertensive emergency. Sodium nitroprusside, a short-acting intravenous vasodilator, has been utilized for a long time, despite only limited available data on its safety and efficacy. Although this limited body of evidence supports its efficacy in reducing BP, this drug should be used with caution, especially in children whose hypertensive crises are commonly secondary to renal disease, because of the accumulation of its toxic metabolites (cyanide and thiocyanate) (5, 39, 40).

Given the risk of nitric oxide toxicity, in literature new drugs are emerging for the use also in the intensive setting in pediatric patients.

Children who present with hypertensive crises frequently suffer from secondary hypertension, and kidney disease is the most common etiology. In our review the main cause of hypertension crises was cardiac surgery (32%), while chronic kidney disease was only the second most frequent cause (28.7%), differently to the literature. However, our results may be biased by the small pool of studies included and the short period considered (7).

In our review, none of the included studies compared oral and intravenous administration of antihypertensive drugs in terms of efficacy and safety. Furthermore, most studies analyzed the efficacy and safety of single antihypertensive drugs. Only Lad et al. (25) performed a comparison between labetalol and sodium nitroprusside, with the limitations of a retrospective study. There are no randomized controlled trials in the literature comparing the use of different antihypertensive drugs in hypertensive crises in children.

The results of our review must be interpreted in the context of its limitations. First, we limited our search to two main databases (PubMed and Cochrane Library). Secondly, unpublished studies and unreported data were not included. Finally, systematic review was not performed.

## Conclusions

The management of pediatric hypertensive emergencies is still an important issue for clinicians, because there is a lack of consensus in literature on the best therapeutic choice.

Beta-blockers seem to be safe and effective in hypertensive crises, more than sodium nitroprusside, although limited data are available. Indeed, calcium-channel blockers seem to be effective and safe, in particular the use of clevidipine during the neonatal age, although limited studies are available. However, further studies should be warranted to define a univocal approach to pediatric hypertensive emergencies.

## Author Contributions

NB, RS, LD, MS, BG, AS, and EZ performed the literature review and drafted the initial manuscript. NB, RS, LD, MN, and DM supervised the literature review, provided critical insight, and prepared the final version of the manuscript. All authors contributed to the article and approved the submitted version.

## Conflict of Interest

The authors declare that the research was conducted in the absence of any commercial or financial relationships that could be construed as a potential conflict of interest.

## Publisher's Note

All claims expressed in this article are solely those of the authors and do not necessarily represent those of their affiliated organizations, or those of the publisher, the editors and the reviewers. Any product that may be evaluated in this article, or claim that may be made by its manufacturer, is not guaranteed or endorsed by the publisher.
